# Growth Factors in Regeneration and Regenerative Medicine: “the Cure and the Cause”

**DOI:** 10.3389/fendo.2020.00384

**Published:** 2020-07-07

**Authors:** Konstantin Yu. Kulebyakin, Peter P. Nimiritsky, Pavel I. Makarevich

**Affiliations:** ^1^Faculty of Medicine, Lomonosov Moscow State University, Moscow, Russia; ^2^Laboratory of Molecular Endocrinology, Institute for Regenerative Medicine, University Medical Research and Education Centre, Lomonosov Moscow State University, Moscow, Russia; ^3^Laboratory of Gene and Cell Therapy, Institute for Regenerative Medicine, University Medical Research and Education Centre, Lomonosov Moscow State University, Moscow, Russia

**Keywords:** growth factor, regeneration, fibrosis, receptor tyrosine kinase, signaling

## Abstract

The potential rapid advance of regenerative medicine was obstructed by findings that stimulation of human body regeneration is a much tougher mission than expected after the first cultures of stem and progenitor cells were established. In this mini review, we focus on the ambiguous role of growth factors in regeneration, discuss their evolutionary importance, and highlight them as the “cure and the cause” for successful or failed attempts to drive human body regeneration. We draw the reader's attention to evolutionary changes that occurred in growth factors and their receptor tyrosine kinases (RTKs) and how they established and shaped response to injury in metazoans. Discussing the well-known pleiotropy of growth factors, we propose an evolutionary rationale for their functioning in this specific way and focus on growth factors and RTKs as an amazing system that defines the multicellular nature of animals and highlight their participation in regeneration. We pinpoint potential bottlenecks in their application for human tissue regeneration and show their role in fibrosis/regeneration balance. This communication invites the reader to re-evaluate the functions of growth factors as keepers of natively existing communications between elements of tissue, which makes them a fundamental component of a successful regenerative strategy. Finally, we draw attention to the epigenetic landscape that may facilitate or block regeneration and give a brief insight into how it may define the outcome of injury.

## Introduction

One of the paradigms in regenerative biology and medicine is that the adult stem cell (SC) is the cornerstone of tissue renewal and regeneration. Its functions are regulated by the nervous system, providing rapid response, and by endocrine stimuli transmitted by hormones, growth factors, and cytokines acting via specific receptors. These systems provide an array of signals required to support tissue homeostasis and repair after damage. Therefore, SC alone is not the optimal object for application in regenerative medicine since it depends on the regulatory circuits of the tissue (much related to the “niche” term) and lacks functional autonomy. Thus, probably the only effective “stem cell therapy” known to rebuild a functional organ from adult SC to date is bone marrow transplantation (1).

The human body possesses an impressive capacity for renewal during the course of life, managing to replace cells in the majority of tissues and organs after their disposal by programmed cell death. At the same time, when reparative regeneration is required to restore structure and function (in its classical definition), *Homo sapiens* is not among the best species to handle this. After minor damage, human tissues with an epithelial component (skin, gut, blood vessels, pancreas, etc.) successfully undergo epimorphic regeneration. However, after major damage occurs, our body has a significant inclination toward fibrosis and hyperplasia of remaining tissue (2). Certain exceptions from that rule exist in the human body, suggesting valid objects to study and supporting the concept that epimorphic regeneration in our bodies is not completely restricted (Table 1).

**Table 1:** Physiological examples of regenerative capacity in humans.

**Outcome after damage**	**Organs, tissues**
Fibrosis (scarring) with hyperplasia to compensate for tissue loss	Majority of parenchymatous organs and tissues in postnatal period (2, 3);
Scar-free epimorphic regeneration	Skin (after superficial injury) and its appendages (nail, hair) (4); imperfect regeneration in distal phalanx (5)
Epimorphic regeneration of structure	Bone (6); endometrium in normal function (7); spleen (8);
Ectopic formation of organotypic structure	Spleen (9); endometrium in endometriosis (10)

Processes of regeneration is mediated by the resident SC identified in most tissues of the adult organism. These cells, such as adipose tissue mesenchymal cells (11), dental-derived (12) or neural SCs (13), and others, play a pivotal regulatory role in both tissue renewal and regeneration after injury. On the one hand, they possess an ability to proliferate and differentiate into a variety of tissue-specific cells, and on the other, they produce tissue-specific matrix and release soluble factors that orchestrate tissue renewal and repair (14, 15). Deep involvement in tissue homeostasis maintenance makes these cells a lucrative object for study and potential application in regenerative medicine (16, 17). Nevertheless, we still have much to find out about the factors and molecular machinery that regulates the functions of these cells (18).

On the molecular level renewal and regeneration are controlled by many classes of soluble bioactive agents. They range from neurotransmitters, short peptides, and chemokines up to growth factors (GFs) – large proteins with a complex process of biogenesis and activation after secretion (19, 20).

One peculiar point is that after damage, the same molecules can drive either regeneration or fibrosis. For example, in *Urodele* amphibians, GFs play a crucial role in limb regeneration, which requires the dedifferentiation of cells, formation of blastema, and subsequent cell re-differentiation that results in limb replacement (21). After amputation, transforming growth factor β (TGF-β), controlling the Smad2/3 axis, and epidermal growth factor (EGF), which regulates transcription factor Yap1 (22), are detected at the site of injury in abundance. These factors are crucial for early cell migration, while inhibition of Smad2/3 or Yap1 signaling was shown to ablate regeneration in axolotl (23, 24). Meanwhile, in mammals, including humans, TGF-β and EGF are among the major factors driving fibrosis after acute damage or in chronic organ disease (25–27). This illustrates that in different species, homologous signal axes driven by similar ligands may results in different outcomes after damage.

Moreover, even within one and the same species, a GF can be pro-regenerative or pro-fibrotic depending on the setting and background of damage that affected the organ. In human, well-known regulators like insulin-like growth factor (IGF), TGFs, and platelet-derived growth factors (PDGFs) drive scar-free regeneration *in utero*, even in late stages of development. In the postnatal period after injury, a similar spectrum of GFs is released via platelet degranulation and produced by immune cells or myofibroblasts, eventually leading to scar formation (28, 29), contrary to full regeneration mediated by the same GFs in human fetus.

The described pleiotropy of GF functions and its putative mechanism shall be discussed in this mini-review further, but for anyone involved in translational studies, at first sight this creates a massive problem that is hard to solve or dissect in search of a solution.

In species with prominent regenerative abilities, activation of GF and their signaling pathways are foundations for epimorphic regeneration, but in human, they become the main drivers of fibrosis and scarring. One potential explanation is that during the evolution of signaling systems from primitive organisms to humans, a crucial structural shift occurred, resulting in loss of regenerative capabilities. However, data from phylogenetic analysis show that systems of GFs and their receptor tyrosine kinases (RTKs) remained highly conserved in animals (30). Thus, species with high and low regenerative capacity utilize a similar molecular “toolkit” to end up with different outcomes.

This situation may severely limit our ability to promote regeneration of tissues via the introduction of GFs or cells producing them, including mesenchymal multipotent stromal cells (MSC), known to act via a repertoire of soluble factors secreted after delivery. However, endogenous SCs also possess high paracrine activity, allowing them to communicate with the tissue environment. At this point, we may raise a number of questions that are crucial for our understanding of this system:

- What was the evolutionary background that led to the development of such a level of pleiotropy in GF function?
- What was the cause of the shift from regeneration to fibrosis as a way to respond to damage?
- Given that GFs and their receptor systems in humans may be obliged to promote fibrosis, can we find a way around this evolutionarily established pattern?

To elaborate on this, we further encourage the reader to re-evaluate the role of GFs beyond the function of individual molecules and present them as mediators that give metazoans the feature of a multicellular structure and define how this structure responds to damage and loss of existing communication between its elements.

## The Growth Factor/RTK Axis in Metazoans Is the Cornerstone of Organism Integrity

During the course of natural history, different methods of intercellular communication have been established (31). In plants and algae, the shift to multicellular life forms was made without a new signaling system, using the same receptor-ligand interactions that their unicellular ancestors had previously had – namely cytokinins and their histidine kinase receptors. While these taxons relied on pre-existing signaling systems and adjusted their function to becomes multicellular, animals made a move to the next level. Indeed, in animals, the emergence of multicellular species was accompanied by a drastic increase in the number of new genes encoding signal transduction proteins compared to protozoans (32).

The multicellularity of metazoans is a feature that cannot be described as a sum of the functions and metabolic needs of individual cells that reside within the organism. In a multicellular organism, despite being “anchored” to a tissue or its specific microanatomical compartment, every cell is permanently receiving multiple, sometimes “contradicting” signals. Making reproducible decisions or interpreting stimuli in such incomprehensible “signal noise” may seem an unsolvable problem. Addressing this challenge prior to forming obligatory multicellularity was required to establish physiological regulation and – basically – subdue functions of individual cells to the needs of the harboring organism.

The majority of elements forming the RTK apparatus evolved long before the emergence of metazoans (33). Different classes of mitogen-activated protein kinases (MAPK) with Ser/Thr activity existed in protozoans and served as downstream effectors of surface-located G-protein coupled receptors (GPCR). The principle of their operation was perfect for unicellular species, as every axis was activated by a specific GPCR and provided fast transduction of a signal evoked by a specific stimulus or condition change (osmosis, starvation, pheromones, etc.) This allowed it to respond rapidly, and these MAPK cascades formed an effective system to monitor the environment and control proliferation in yeast and other protozoans, providing fast and unambiguous signals.

As signal complexity increased, rising ambiguity was resolved by a new MAPK class – Ser/Thr + Tyr protein kinase (MAPKK). This introduced a new mechanism of MAPK activation via double sequential phosphorylation (34), which allowed the whole cascade to acquire a short-term “memory” (35). In this case, the first stimulus primes the cascade by phosphorylation, and for a while, the cell becomes responsive to the second stimulus. This created an opportunity for interference or integration of different incoming stimuli, which later became the basis for the amplification characteristic for GF signaling (36, 37).

Further, as communities of unicellular organisms became more complex, this system evolved to mediate intercellular communication by secreted factors, including the ancestors of modern GFs. At some point, the final change required for a shift to an obligatory multicellular structure was made. Control of MAPK phosphorylation was “diverted” from GPCRs and granted to a newly emerging class of receptors – RTKs. Briefly, multicellular organisms constructed a “finishing block” and built it over an existing MAPK signal transduction system, reassigning the activator role to RTKs.

At that point, one may still question the rationale for pleiotropy of GFs and their ambiguous function, where one molecule may have opposite functions (e.g., be pro-fibrotic and pro-regenerative) in different settings. In contrast to hormones, where specialization of signals was achieved by expanding the diversity of molecules with a unique signal function, in the GF/RTK axes, signal transduction and processing has become the basis for efficient communication between cells in metazoans.

Indeed, an organism with over 200 tissue types requires a universal means of communication that can be discerned by different specialized cells. Having 200 cell-specific GFs and every other cell expressing some of the 200 GF-specific receptors was definitely a redundant, non-optimal solution, also excluding the ability of the cell to process multiple signals or amplify them. That became the rationale for having a limited number of GF families but generating cellular processing machinery that can process multiple incoming signals. Thus, GF pleiotropy may have appeared as a means to transduce as much information as possible using a limited number of molecules, and RTKs are used to decipher these messages. This allows the “noise” to be filtered and a sum of stimuli to be accumulated, interpreted as instructions, and transferred to the cell's machinery.

In regeneration, the crucial function of GFs is the establishment of correct intercellular communications. It does not exclude conventionally acknowledged activities: driving successful acquisition of function/phenotype by individual cells (e.g., SC differentiation) or cell division. This suggestion complies with another observation – as organism complexity increased over the course of evolution, regenerative capacity tended to decrease. This may reflect a well-known engineering principle that the more the complex system destroyed by damage, the harder is the task of its reconstruction (11).

Viewing tissue repair process in this way provides an explanation of why the same GFs that support tissue homeostasis and renewal are the drivers of fibrosis after injury. At the site of damage, huge amounts of GFs (TGF, PDGF, EGF, etc.) are released from platelets along with local production, creating a very multifaceted signal. In most human tissues, after significant damage, local stromal cells use their RTK to process this initially incomprehensible signal and drive fibrosis. In the described case, GFs may be expected to launch restoration of structure but they fail to dictate a regenerative program despite abundant presence at the site of injury. At the same time, these early-stage GFs are absolutely indispensable – even a short delay or inhibition of RTK activation results in serious distortion of the regenerative process (38). Thus, in acute phase of response to injury become “the cure and the cause,” and form a physiological link that cannot be easily influenced by chemicals or other means without consequences for outcome.

## The Epigenetic Landscape Modulates the Effects of the Growth Factor/RTK Axis in Regeneration

Signaling pathways from GF-triggered RTKs are well-conserved within the Animal Kingdom, raising the question of what has changed, altering human tissues and communication patterns and creating an inclination toward fibrosis compared to other species. A possible answer is that our epigenetic landscape is responsible for the cellular effects of RTKs. The latter create a “slower” signaling pathway than ion channels or GPCRs, but RTKs exert signaling via nuclear trafficking of effector protein kinases and activation/repression of transcription factors. Their ability to modulate the expression of genomic sequences is highly dependent on what sites of DNA are open for interaction. At this point we cannot ignore the epigenetic landscape, which contributes to the pleiotropy of GF/RTK signaling effects in regeneration.

For example, Sonic hedgehog (Shh) is crucial for both development and regeneration. Regulation of its gene expression provides a good example of the connection between the epigenetic profile and the regenerative capacity of an organism. During limb development or regeneration, Shh is expressed in the posterior region, where it is responsible for anterior/posterior polarity and takes part in the formation of digits. The expression of Shh gene is controlled by a specific enhancer, MFCS1 (39). In *Xenopus*, this enhancer displays low methylation at the tadpole stage, which is known to regrow amputated limbs by the formation of blastema. However, after metamorphosis to froglets, MFCS1 becomes highly methylated, which corresponds with a loss of regenerative potential at this stage. Froglets are unable to perform complete limb regeneration but instead form a spike-like cartilage structure. In contrast, in axolotl capable of complete limb regeneration during their entire lifespan, the MFCS1 enhancer remains hypomethylated. This methylation is tightly linked with the expression of Shh gene, and high levels of methylation of MFCS1 prevent Shh expression (40). These findings link the regenerative capacity of the organ with the epigenetic status of cells within it.

**Figure 1:**
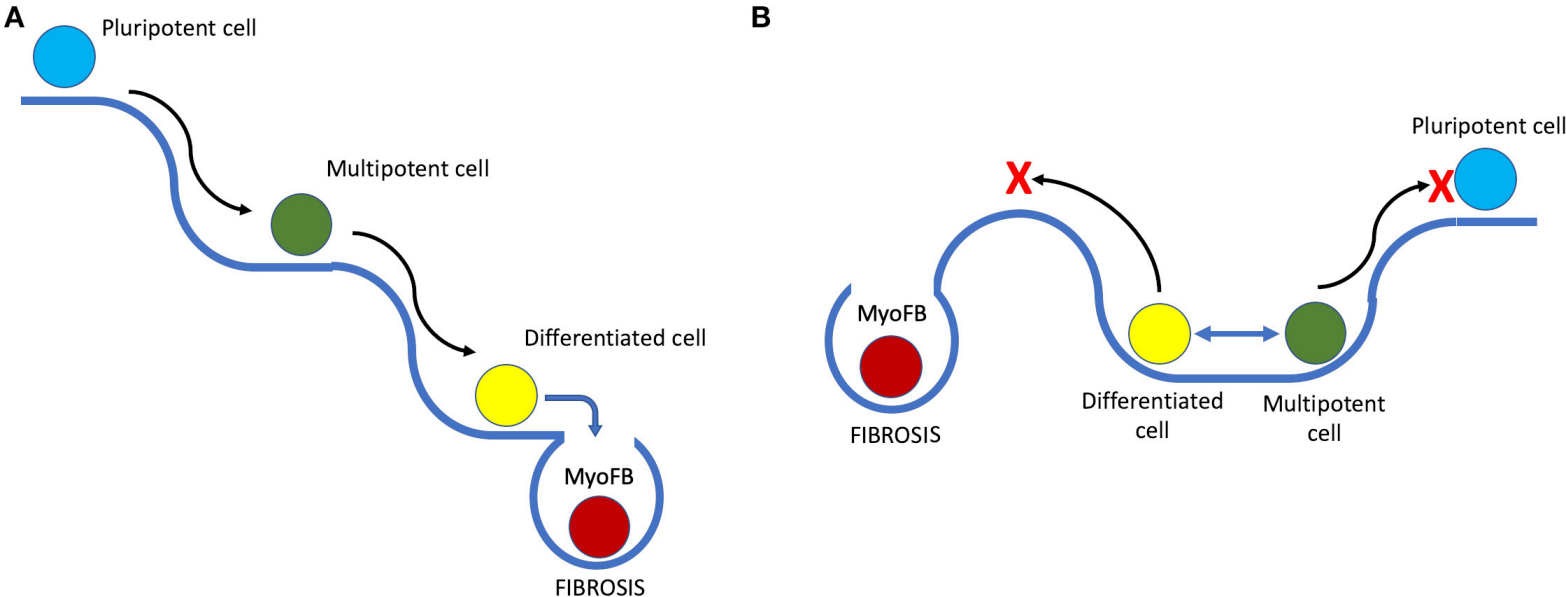
Putative scheme of the epigenetic landscape in species with high and low regenerative capacities and its influence on cell fate. **(A)** Epigenetic landscape in species with low regeneration. Black arrows represent differentiation, and slopes indicate low probability of phenotype reversion or dedifferentiation; blue arrow highlights the moment when, after damage, a myofibroblast (MyoFB) “falls off the cliff,” and an irreversible cell fate decision is made, followed by scarring. **(B)** Depiction of a different landscape that favors phenotype change and transient dedifferentiation with limited stemness acquisition (blue 2-headed arrow on the plateau). The red cross marks potential restriction on both pluripotency acquisition and fibrosis imposed by the epigenetic landscape, reducing probability of an unfavorable cell fate decision after damage.

It is known that during regeneration in amphibians, cells at the site of injury undergo dedifferentiation to form a blastema (41) and later differentiate into new functional tissue (42). However, multiple studies have shown that unlike the formation of induced pluripotent cells that lose all their cell lineage-specific epigenetic markers, blastema cells derived from bone, muscle, or dermal cells, contribute mostly to the formation of the respective cell type during regeneration (43). After dedifferentiation, cells in regenerating animals retain a lineage-specific epigenetic profile – a so-called cell lineage memory. For example, bone-derived blastema cells regenerate into bone but not muscle or dermal cells. This means that the dedifferentiation that precedes regeneration is limited, and cells gain plasticity for active proliferation and tissue formation rather than true pluripotency (Figure 1).

If looked at from the standpoint of differentiation potential, fibrosis is an opposite condition to formation of blastema. By excessive matrix deposition fibrosis prevents taxis and migration of terminally differentiated cells and blocks their potential proliferation. This reaction may seem as counter-evolutionary - complete restoration of tissue function after injury is a major advantage. However, when our ancestors moved from the sea to the surface, they faced hyper-oxidative conditions in this new environment (44). A high level of oxygen may interfere with the regenerative process associated with cell dedifferentiation, which requires decompactization of DNA and results in its increased sensitivity to damage by reactive oxygen species (ROS) (45).

Such, organisms have more compact chromatin to prevent dedifferentiation or very active proliferation and eventually “patch up” the wound by connective tissue. Thus, fibrosis may reflect the properties of a more restrictive system that maintains the genomic stability of the cells.

A moment when a dramatic shift of the epigenetic status of the genome occurs is present in most land species, including humans, namely the moment of delivery when the organism leaves the hypoxic aqueous environment of the uterus/egg and is exposed to a highly stressing atmospheric level of oxygen (46, 47). ROS-mediated DNA damage is quickly repaired, yet epigenetic modifications are accumulated, changing the expression of hundreds of genes encoding proteins and regulatory RNAs of different classes (48, 49). These changes may somehow resemble the water-to-land transition, yet a newborn has several days to adapt to the new environment (49). We still may speculate on what was the driving force and why fibrosis originated in land animals. We believe that after moving to atmospheric oxygen levels, species that tried to regenerate the same way that they did an aqueous environment were putatively eliminated.

There is a hypothesis that fibrosis might have been protective against negative consequences of ROS impact and epigenetic distortions in these early land animals to prevent cancer (50). Unfortunately, a scar is non-receptive to normal tissue elements as well (blood vessels, stromal cells, nerve terminals, SC, and parenchyma), which resulted in a side effect of this adaptation, namely a huge decline in ability to regenerate after damage.

## Conclusion

Overall, we may conclude that GFs are an evolutionary established unique system that provides tissue formation in development and then via RTKs and their signaling axes supports homeostasis, cell integration, and tissue renewal. However, after damage, they may become “the cure and the cause,” as positive and negative outcomes are mediated by the same GFs depending on species or specific tissue within the organism.

We highlighted the epigenetic landscape as a putative reason why highly conserved GFs and RTK pathways may fail to induce full-scale regeneration in species known to undergo fibrosis (including humans) and be the driving force of regeneration in others. Investigation of epigenetic regulation in connection with regeneration in humans might open a new field and provide targets for therapies that will rely not on ligands but on their eventual targets – genomic sequences and regulatory mechanisms that define cell fate in health and disease.

## Author Contributions

PN wrote parts of the manuscript. KK wrote parts of the manuscript and proofed. PM idea of the manuscript, prepared the figures, proofed provisional versions, and approved submission. All authors contributed to the article and approved the submitted version.

## Conflict of Interest

The authors declare that the research was conducted in the absence of any commercial or financial relationships that could be construed as a potential conflict of interest.
